# Prevalence of Drug Resistance Mycobacterium Tuberculosis among Patients Seen in Coast Provincial General Hospital, Mombasa, Kenya

**DOI:** 10.1371/journal.pone.0163994

**Published:** 2016-10-06

**Authors:** Ida Pam Ombura, Noel Onyango, Susan Odera, Florence Mutua, Joshua Nyagol

**Affiliations:** 1 Department of Medical Microbiology, University of Nairobi, Nairobi, Kenya; 2 Department of Human Pathology, Unit of Immunology, University of Nairobi, Nairobi, Kenya; 3 Department of Clinical Medicine and Therapeutics, Unit of Medical Oncology, University of Nairobi, Nairobi, Kenya; Fundació Institut d’Investigació en Ciències de la Salut Germans Trias i Pujol, Universitat Autònoma de Barcelona, SPAIN

## Abstract

**Background:**

Although prevention and control of spread of multi-drug resistant tuberculosis strains is a global challenge, there is paucity of data on the prevalence of DR-TB in patients diagnosed with TB in referral hospitals in Kenya. The present study assessed patients’ characteristics and prevalence of drug resistant TB in sputa smear positive TB patients presenting to Coast Provincial General Hospital (CPGH) in Mombasa, Kenya.

**Methods:**

Drug resistance was evaluated in 258 randomly selected sputa smear TB positive cases between the periods of November 2011 to February 2012 at the CPGH-Mombasa. Basic demographic data was obtained using administered questionnaires, and clinical history extracted from the files. For laboratory analyses, 2mls of sputum was obtained, decontaminated and subjected to mycobacteria DNA analyses. Detection of first line drug resistance genes was done using MDRTDR plus kit. This was followed with random selection of 83 cases for second line drug resistance genes testing using Genotype MDRTBsl probe assay kit (HAINS Lifesciences, GmbH, Germany), in which ethambutol mutation probes were included. The data was then analyzed using SPSS statistical package version 19.0.

**Results:**

Male to female ratio was 1:2. Age range was 9 to 75 years, with median of 30 years. New treatment cases constituted 253(98%), among which seven turned out to be PTB negative, and further grouped as 4 (1.6%) PTB negative and 3(1.1%) NTM. 237(91.7%) new cases were fully susceptible to INH and RIF. The remaining, 8 (3.1%) and 1(0.4%) had mono- resistance to INH and RIF, respectively. All the retreatment cases were fully susceptible to the first line drugs. HIV positivity was found in 48 (18.6%) cases, of which 46(17.8%) were co-infected with TB. Of these, 44 (17.1%) showed full susceptibility to TB drugs, while 2 (0.8%) were INH resistant. For the second line drugs, one case each showed mono resistance to both and FQ. Also, one case each showed drug cross poly resistance to both ETH and FQ, with second line injectable antibiotics. However, no significant statistical correlation was established between TB and resistance to the second line drugs p = 0.855.

**Conclusion:**

The findings of this study showed the existence of resistance to both first and second line anti-tubercular drugs, but no MDR-TB and XDR-TB was detected among patients attending TB clinic at CPGH using molecular techniques.

## Introduction

An estimated 29% of the world’s TB cases occur in the African Region, where high HIV prevalence has been known to drive the TB epidemic [1]. Kenya ranks fifteenth among the twenty two high burden TB countries in the world, where HIV prevalence is also estimated at 5.6–6.3% nationally, with significant regional variation[2,3]. According to the WHO 2014 report, 38% of the TB patients in Kenya were co-infected with HIV in 2013 [1].

Although the development of drug resistant TB strains and consequent treatment failure is a common clinical scenario in TB disease, and associated with high mortality rates, information on drug resistance as well as both multi-drug and extensively drug resistant tuberculosis is currently very scanty in Sub-Saharan Africa, Kenya inclusive [4]. In addition, only a few isolated cases of MDR-TB in Kenya have been reported [5]. This is attributed to very low rate of detection, as the diagnostic methods available in the public health facilities are still largely based on sputum slide smear microscopy [6,7]. This technique has low sensitivity and lacks specificity for susceptible tuberculosis mutant strains that commonly confer resistance to the approved MTB regimens [8].

Nevertheless, the effective diagnosis and treatment of Mycobacterium tuberculosis has remained a global health challenge for many years. This is further complicated with the frequent co-existence of epidemics of TB and human immunodeficiency virus (HIV), as well as the increasing prevalence of multidrug-resistant TB (MDRTB) [8]. In fact, in 1993, the WHO declared the disease a global emergency and public health concern as a result of the HIV/AIDS pandemics [9]. In addition, other studies have also reported that the detection of NTM, a common opportunistic infection in TB-HIV/AIDS is often missed due to the overlapping clinical manifestations of MTB. Also, the disseminated NTM disease only occur in severe immune suppression, and their treatment considered to be different to that of TB [10,11].

Development of Drug resistant TB strains are commonly associated with high mortality rates, especially with Drug resistant tuberculosis (DR-TB), Multi-drug resistant tuberculosis (MDR-TB), extensively drug resistant tuberculosis (XDR-TB), and total drug resistant tuberculosis (TDR-TB) [12]. However, in most cases MDR-TB develops during treatment of fully drug susceptible TB, due to interruption of the treatment therapy or inappropriate treatment choice. The resulting event is bacterial mutation and generation of genotypes that are tolerant to the toxic effects of the commonly used anti-mycobacterial drugs [13].

Since antimicrobial resistance invariably follows the introduction of new drugs, appropriate drug-susceptibility testing assays are therefore needed to detect resistance and tailor treatment regimens that contain new agents [14]. Given that phenotypic drug-susceptibility testing is slow, technically demanding, and, in some cases, unreliable, rapid molecular techniques have been used in TB drug resistance assays [15]. It is in this context that the present study used molecular technique of Hains Lifescience for the detection of DR-TB, MDR-TB and XDR-TB in patients who had sputum smear positive mycobacterium tuberculosis bacillus (MTB) at the Coast General Hospital in Mombasa Kenya, between January and September, 2012. The findings were then correlated with demographic and clinical characteristics of individual patients for significant associations.

## Materials and Methods

### Study design

This was a descriptive cross sectional study, carried out at Coast General Referral Hospital Mombasa, Kenya between January and September, 2012. Approval to conduct the study was obtained from the Kenyatta National Hospital/University of Nairobi (KNH/UON) Ethical and Research Committee (ERC) and Coast Provincial General Hospital administration, prior to the commencement of the study. The demographic and clinical data were obtained during the routine clinical check-up, after obtaining signed informed consent from the study cases. The cases which were reported as sputum smear positive for TB were then subjected to molecular analyses for drug resistance strains at the medical microbiology department of the University of Nairobi.

### Study population

The study population involved 258 sputum smear (SM) TB positive patients from direct fluorescent staining and microscopy, using auramine and rhodamine stains, at the microbiology section of the CPGH. These were eligible patients who met the inclusion criteria, and voluntarily gave signed informed consent to participate in the study.

### Demographic and clinical data collection

Upon obtaining signed informed consent, the patients were interviewed using structured questionnaire to collect data on their demographic characteristics and medical history. In addition, data generated from the patients’ laboratory request forms as well as files were recorded into the data collection forms, and subsequently entered into a database.

### Specimen collection and laboratory analyses

All the sputa from patients attending routine TB clinic at CPGH that tested positive for tuberculosis using fluorescent microscopy (FM) were labeled with unique corresponding study number and stored in the refrigerator at 4°C for further analysis. Thereafter, approximately 2mls of the sputum from each case was aliquoted separately into a clean labeled falcon tube and decontaminated/digested using 4% sodium hydroxide, according to modified PETROFF’S procedure [16]. The aliquots were stored at -20°C, before being transported to the University of Nairobi, mycobacterium molecular laboratory in accordance with the International Air transport Association (IATA) protocol for clinical infectious specimens for TB strains’ DNA analyses.

### Genotyping for MTB drug resistance

For drug resistance testing, DNA was extracted each sample, amplified and hybridized with probes on an immobilized phase, using the Hains Lifesciences manual for Genotype MDRTB® plus, and MDRTBsl plus kits for first and second line drug resistance respectively. Sputa specimens which showed inconsistent bands on the MTBDR®*plu*s strip, and/or no MTB control band underwent repeat PCR and hybridization. All of the 258 smear positive clinical specimens were directly subjected to Hains Lifescience (GmbH, Germany) assays, MTBDR® plus for first line drugs, and subsequently 83 samples were randomly selected and evaluated using MTBDRsl for second line drugs.

Analyses with Hains Life sciences genotype test kits were done to screen for mutant MTB strains and predict mutational gene patterns associated with drug resistance. The MTBDR®plus assay was used to analyze genes associated with INH and RIF resistance, the main first line anti-TB drugs, and MTBDRsl assay for the ethambutol and second line drug resistance gene patterns, as previously described [17]. In brief, for the MTBDR®plus assay, 500μl of sputa positive clinical samples was decontaminated and block heated at 95°C for 20 minutes, followed by sonification for 15min and centrifugation at 13,000 revolutions per minute for 5 minutes to extract the mycobacteria cell DNA. Then, 5μl of the supernatant was added to 45μl of PCR master mix. The mixture was then loaded into a programmed real time thermocycler for amplification of the drug resistance-determining region (DRDR) of the target gene, with addition of biotinylated primers (3' TGA CCTGAAAAGAC 5'). The thermocycling conditions involved first step at 95°C for 15 minutes, followed with twenty cycles at 95°C for 30 seconds, 65° for 2 minutes; thirty cycles at 95° for 25 seconds, 50°C for 40 seconds, 70°C for 40 seconds and elongation step of one cycle at 70°C for 8 minutes. The amplicons were then stored at 4° C in the refrigerator.

For MTBDRsl assay, 500μl of the decontaminated sputum of positive clinical samples were block heated at 95°C for 20 minutes, followed by sonification for 15min and centrifugation at 13,000 revolutions per minute for 5 minutes, to extract the mycobacteria cell DNA. This was followed with addition of 5μl of the supernatant to 45μl of PCR master mix. The mixture was then loaded into a programmed real time thermocycler for amplification of the drug resistance-determining region of the gene, with addition of biotinylated primers. The thermocycling conditions involved first step of heating 95°C for 15 minutes (1 cycle), followed with ten cycles at 95°C for 30 seconds, 58°C for 2 minutes; thirty cycles at 95°C for 25 seconds, 53° C for 40 seconds, 70°C for 40 seconds and elongation step of one cycle at 70°C for 8 minutes. The amplicons were then stored at 4°C in the refrigerator.

Post-PCR analyses for the drug resistance genes were done using hybridization processes. This involved the first step in which 20μl of denaturation solution was added to 20μl of DNA PCR products, and mixed by action of pipetting the mixture up and down. The mixture was then incubated at room temperature for 5 minutes. After incubation, 1ml of hybridization solution was added to each tray well containing the denatured DNA and mixed by tilting the mixture in the tray up and down using a twin incubator for 30 minutes at 45°C.

The Hains Lifesciences test kits have probes embedded in the strips that result in attachment of complementary DNA sequences. Primers used in the amplification process are also biotinylated, and when corresponding amplicons are correctly subjected to prescribed protocol conditions, the complementary sequences are visible as bands on the strips. These bands are further interpreted to define positive diagnosis, or absence of susceptible TB, MTBC (Mycobacterium TB Complex), NTM (Non-Tuberculosis Mycobacteria), any mono-drug resistant TB, MDR-TB, or XDR-TB as the case may be [18, 19]

Therefore, for our experiment, the strips were labeled with the study identifiable number using DNA strip marker. The labeled strips were placed in corresponding trays containing samples with denatured DNA, and incubated for 30 minutes at 45°C in the Twincubator. This was followed by complete aspiration of the mixture. Then, 1 ml of STR (fixing and washing solution) was added to each tray, and incubated again for 15 minutes in the Twincubator. The STR solution was aspirated, and 1 ml of RIN (rinsing solution) added to each tray and further incubated for 1 minute at room temperature in the Twincubator, before aspirating the whole quantity of RIN from the well. DNA strips were then removed from each tray, and air dried on absorbent paper. Using a transparent cellophane paper, the dried strips were attached onto a result sheet labeled with each sample’s identifiable number and interpreted using the respective interpretation charts provided with the test kit.

MTBDR*®plus* gene probe test strip was used to identify INH and RIF mutant / drug resistant strains, and contained 27 reaction zones (GenoType MTBDRplus version; http://www.hain-lifescience.de/en/products/microbiology/mycobacteria/tuberculosis/genotype-mtbdrplus.html).

This included 6 control signal probe bands such as conjugate control (CC), amplification control AC, *M*.*tuberculosis* complex (MTB) control (TUB), rpoB gene amplification control (rpoB), katG gene amplification control (katG) and inhA gene (inhA) amplification control. Also included are 21 signal probe bands with both wild and mutant probes for the drug susceptibility testing (RIF and INH drug target genes). For a test to be reported valid, the six control bands were observed at their designated sites, and drug resistant strain was identified by missed probe in the wild type probe, or addition of mutant probe. For example INH mutant strains resistance was interpreted as presence of INH gene locus, missing wild type probe band or having mutant probe band.

MTBDR*sl* gene probe test strip was used to identify ETH, FQ, KAN, AMK, CAP and VIO drug resistant mutant strains, contained in 22 reaction zones (GenoType MTBDR*sl*; http://www.hain-lifescience.de/en/producs/microbiology/mycobacteria/tuberculosis/genotype-mtbdrsl.html). This included 6 control signal probe bands such as, conjugate control (CC), amplification control (AC), *Tuberculosis* complex control, gyrA gene locus control, rrs gene locus control and embB gene locus control. Also included are 16 signal probe bands with both wild and mutant probes for drug susceptibility testing, as observed in drug target gene interpretation chart. For a test to be reported valid, six control bands were observed at their designated sites of CC, AC, MTB complex, gyr A, rrs, and embB locus). Drug resistant strain was identified by missed probe in the wild type probe, or addition of mutant probe. For example, FQ mutant strains resistance was interpreted as presence of gene locus, missing wild type probe band but having mutant probe band. Whereas ETH and aminoglycoside resistance (cross poly-resistant) was interpreted as presence of gene locus for both drugs, with missing wild type probe band and presence of mutant probe band for both drugs.

## Statistical Analysis

Data was imported to SPSS version from excel spread sheet, and analyzed using SPSS v.19 (SPSS Chicago Illinois, USA). Demographic characteristics such as age and sex were summarized into means and percentages. The Pearson Chi-Square analysis was performed to test significant association between age, sex, patient category and drug resistance. Values of p < 0.05 were considered statistically significant.

## Results

### Demographic details and clinical data

From the study cases, males were 174 (67.4%), while the females were 84 (32.6%). The age of the patients studied ranged from 9 to 75 years, with median age of 30 years. Notably, out of the 258 cases, 48 (18.6%) patients were HIV positive, 97 (37.6%) were HIV negative, while 113 (43.8%) had unknown HIV status. In terms of patient categorization, 253 (98.1%) patients were new cases, 1 (0.4%) follow-up, 1 (0.4%) defaulter, and 3 (1.2%) were relapse cases. In addition to the above categorization, 47 of the HIV positive cases were classified as new cases, while one was a follow up case. Among the HIV negative cases, 96 were new, while one case was a relapse. For those with unknown HIV status, 110 were new cases, while 2 and 1 case each were relapse and defaulter cases, respectively (**Table 1**).

**Table 1:** First line drugs susceptibility testing stratified by gender, age, study type and HIV status.

Characteristic	Category	MTB negative n(%)	Fully susceptible n(%)	INH resistant n(%)	RIF resistant n(%)	Total
Gender	Male	4(1.6)	164 (63.6)	5 (1.9)	1 (0.4)	174 (67.4)
	Female	3(1.7)	78 (30.2)	3 (1.2)	0 (0.0)	84 (32.6)
Age range	1–10 yrs	0 (0.0)	7 (2.7)	0 (0.0)	0 (0.0)	7 (2.7)
	11–20 yrs	1 (0.4)	30 (11.6)	0 (0.0)	0 (0.0)	31 (12.0)
	21–30 yrs	1 (0.4)	102 (39.5)	2 (0.7)	1 (0.4)	106 (41.1)
	31–40 yrs	5 (1.9)	66 (25.6)	3 (1.2)	0 (0.0)	74 (28.7)
	41–50 yrs	0 (0.0)	25 (9.7)	2 (0.7)	0 (0.0)	27 (10.5)
	51–60 yrs	0 (0.0)	10 (3.9)	1 (0.4)	0 (0.0)	11 (4.4)
	61–70 yrs	0 (0.0)	1 (0.4)	1 (0.4)	0 (0.0)	1 (0.4)
	71–80 yrs	0 (0.0)	1 (0.4)	1 (0.4)	0 (0.0)	1 (0.4)
Study type	New cases	7 (2.7)	237 (91.7)	8 (3.1)	1 (0.4)	253 (98.1)
	Follow up	0 (0.0)	1 (0.4)	0 (0.0)	0 (0.0)	1 (0.4)
	Relapse	0 (0.0)	3 (1.2)	0 (0.0)	0 (0.0)	3 (1.2)
	Defaulters	0 (0.0)	1 (0.4)	0 (0.0)	0 (0.0)	1 (0.4)
HIV status	Negative	96 (37.2)	89 (35.0)	5 (1.9)	1 (0.4)	96 (37.2)*
	Positive	1 (0.4)	44 (17.3)	2 (0.8)	0 (0.0)	48 (18.6)*
	Unknown	113 (43.8)	109 (42.6)	1 (0.4)	0 (0.0)	113 (43.8)*

*1 HIV positive was found to be NTM; 2 HIV negative and 3 cases of unknown HIV status were negative for first line drug test.

### MTB confirmation and susceptibility testing for first line drugs

With the MTBDR®plus assay for MDR-TB, all the 258 cases were subjected to first line drug susceptibility test. Out of these, 251 were confirmed to be positive for MTB, while 4 isolates had MTB negative results, despite being initially identified as Acid Fast Bacillus positive with fluorescent microscopy technique. In addition, three cases which were initially reported as MTB complex had non tuberculosis mycobacterium (NTM). In terms of susceptibility, a total of 242 cases were fully susceptible to the first line drugs, while eight cases were resistant to INH. Resistance to RIF was detected in only one case (Table 1). Pearson Chi-Square analysis showed no significant statistical correlation between MTB drug resistance and gender, *p* = 0.320.

### Age categorization and susceptibility testing for first line drugs

The study cases were further categorized according to age range and first line drug susceptibility test. Age range of 21–30 had highest incidence of TB (41.1%) followed by age range of 31–40 years (28.7%) and 11–20 years 31(12%). Overall, 9 (3.5%) isolates had drug resistant genes to FLD (INH, RIF). Of these 8 (3.1%) had INH resistance, 1 (3.1%) RIF resistance. Drug resistant TB was found from the age range of 21–60 years, of which 2 (0.8%) cases within 21–30 years had drug resistance to INH, while 1(0.4%) cases was resistant to RIF. In addition, 2(0.8%) cases aged between 41–50 years and 1(0.4%) aged 51–60 years also had INH drug resistance (Table 1).

### Patients’ stratification and susceptibility test to first line drugs

The total 258 study cases were stratified as either new cases that had not been put on anti-TB treatment, follow-up, relapse or defaulter cases. Out of these, 253 patients were new cases, from which 8 (3.1%) cases showed resistance to isoniazid, while only one case (0.4%) was resistant to rifampicin. Three out of the total number of cases were found to be relapse cases. In addition, one was a defaulter, while one was a follow up case. All the five cases were fully sensitive to first line drugs (Table 1). However, no significant statistical correlation between the categorization of the cases and first line drug resistance testing was established, *p* = 1.0.

### HIV/AIDS status and susceptibility to first line drugs

From the 48 cases with both TB and HIV co-infection, 44 (91.6%) had drug susceptible MTB gene patterns for first line drugs, while 3 (6.2%) cases showed resistance to INH. Resistance to RIF was, however, not detected. Furthermore, one isolate had NTM. Of the 114 cases with unknown HIV status, 109 (42.2%) were fully susceptible to first line drugs, 1 (0.4%) had INH resistance gene, and no RIF resistance was established. NTM was detected in 2 (0.8%) of the cases in this category.

Among the 96 (37.2%) HIV/AIDS negative cases, 89 (34.1%) were fully susceptible to first line drugs, 5 (1.9%) cases showed resistance gene patterns to INH, while 1 (0.4%) case was resistant to RIF. Nonetheless, 2 (0.8%) out of the 96 isolates were MTB negative. However, Pearson Chi-Square analysis showed no significant statistical correlation between HIV status and MTB drug resistance, *p* = 0.968.

### Susceptibility testing for second line drugs

For second line drug susceptibility testing, 83 cases were randomly selected from the MTBDRplus positive study cases and categorized in age range against drug susceptibility test. It is important to point out that ETH, although is a first line drug, was included in the MTBDR*sl* gene probe kit, which tests for susceptibility to second line drugs. Therefore, out of the 83 cases, 74 (89.2%) were fully susceptible to both SLD and ETH, 4 (4.8%) isolates showed drug resistance to SLD and ETH, while five cases had invalid results (Table 2).

**Table 2:** Second line drug susceptibility testing stratified by age, study type and HIV status.

Characteristics	Category	MTB negative n(%)	Incomplete Test results	FS n(%)	ETH resistant n(%)	FQ resistant n(%)	FQ,+inj Res n(%)	ETH = inj Res n(%)
	11–20 years	0	0	13 (15.7)	0	0	0	0
	21–30	0	0	47 (56.6)	0	0	1 (1.2)	1 (1.2)
	31–40	0	2 (2.4)	14 (16.9)	1 (1.2)	1 (1.2)	0	0
	41–50	0	2(2.4)	2 (2.4)	0	0	0	0
	51–60	0	0	0	0	0	0	0
	61–70	0	1 (1.2)	0	0	0	0	0
	71–80	0	0	0	0	0	0	0
Study type	New cases	0	5(6.0)	0	1 (1.2)	1 (1.2)	1 (1.2)	1 (1.2)
HIV status	Negative	0	2 (2.4)	27 (32.5)	0	1 (1.2)	0	1 (1.2)
	Positive	0	0	15 (18.1)	0	0	0	0
	Unknown	0	3 (3.6)	32 (38.6)	1 (1.2)	0	1 (1.2)	0

Note: Incomplete results were those cases that had technical problems during the analyses.

### Age categorization and susceptibility testing for second line drugs

In terms of age range the 74 fully susceptible cases had distribution of 13(15.7%), 47(56.6%) and 14(16.9%), in the age ranges of 11–12, 21–30, and 31–40 years, respectively. The age range of 21–30 had 2 cases of cross resistance to ETH/KAN, AMK, CAP and FQ/KAN, AMK, CAP while the age range of 31–40 years had two cases of mono-resistance to ETH and FQ (Table 2)

### Patients’ stratification and susceptibility test to second line drugs

All randomly selected 83 from 258 isolates for SLD and ethambutol resistance testing were all new cases, of which 74 were fully susceptible, while one case each showed mono-resistance to fluoroquinolones and ethambutol. In addition, one case was resistant to combination of fluoroquinolones and injectable antibiotics (CAP, VIO KAN, AMK), while one case was resistant to combination of ethambutol and injectable antibiotics (CAP, VIO KAN, AMK). Five clinical samples had incomplete findings during the analysis (Table 2).

### HIV-1 status and susceptibility to second line drugs

All the 15 (18.1%) HIV/AIDS cases were found to be fully susceptible to the second line drugs and ethambutol. From the 31 (37.3%) HIV negative cases, 27(32.5%) were fully susceptible, 1(1.2%) mono resistant to FQ, I (1.2%) cross resistance to ETH /combination with ETH / KAN, AMK, CAP. However, 2 test reports in this category could not be validated. Of the 37 (44.6%) unknown HIV/AIDS cases, 32 (38.2%) had full drug susceptibility, 1 (1.2%) mono resistant to ETH, and 1(1.2%) cross resistant to FQ with combination of KAN, AMK, CAP. However 3 test reports in this category could not be validated (Table 2).

## Discussion

This study aimed at documenting TB resistance patterns as well as patients clinical characteristics in a referral hospital in Kenya. Although Sub-Saharan Africa, Kenya included, has the burden of both high TB incidence and HIV prevalence rates in the world, information on the extent of drug resistant tuberculosis is very limited [20]. This is partly due to poor laboratory facilities, surveillance mechanisms, as well as reporting procedures [21]. HIV infection, in particular, is known to be associated with higher risk of TB and increases the lifetime risk of progression from *Mycobacterium tuberculosis* infection to active disease [22]. Similarly, Tuberculosis (TB) is one of the most common opportunistic co-infections among HIV-infected individuals, particularly in resource-limited settings, with drug resistance resulting from inappropriate treatment [23].

Nevertheless, it has been reported in other studies that the first key intervention for reducing the burden of HIV-associated TB is HIV testing for TB patients. In fact, the WHO report of 2014 indicated that in 2013, 48% of TB patients globally had a documented HIV test result. In the African Region, 76% of TB patients were also indicated to know their HIV status [24]. This is contrary to our findings which showed that 43% of the studied population, even though were either fully susceptible or only resistant to isoniazid, had unknown HIV status. Equally, the majority of the patients in this study were males, most likely attributed to high reporting of the cases. This is supported by previous studies documenting about 60% of TB cases and deaths in men. However, the burden of TB disease among women has also been indicated to be high, a large proportion who are also HIV positive [25].

Currently, MDR-TB poses a great threat to global TB control programs, a factor that is aggravated in resource limited and low-income countries by inadequate availability of prompt diagnostic and treatment options [26]. In Kenya, efforts exist at local and national levels to strengthen TB control, but there is a concern with isolated reporting of the emergence of multidrug resistant TB (MDR-TB) and extensively drug resistant TB (XDR-TB cases [24]. In addition, the diagnosis of MDR-TB in HIV-infected individuals has been reported to be challenging, as the classical symptoms of PTB may not be as evident as in HIV negative patients [27]. Also, HIV positive patients are sometimes negative on smear alone, and thus the presentations of TB among these patients may be over-looked and attributed to the HIV infection [28].

Although the rapid spread of MDR-TB among HIV-positive patients has been associated with impaired ability of the immune system to contain the MDR-TB bacilli in these patients, the adverse events from lifelong treatment of HIV with antiretroviral therapy (ART) coupled with side effects from MDR-TB drugs make the management and outcomes of MDR-TB in HIV co-infected patients very challenging [29]. However, World Health Organization (WHO) recommends the use of culture-independent molecular tools, such as Xpert MTB/RIF to rapidly identify MDR-TB when drug resistance is suspected, and especially in cases of HIV/TB co-infection [30]. This enables rapid diagnosis, early initiation of adequate treatment, patient follow-up, prevention of the spread of drug-resistant TB, and disease surveillance [29].

In the present study, use of genotype MDRTR plus and MDRTBsl assay kits (Hains Lifescience, GmbH, Germany) which target resistance-conferring mutations showed no difference between HIV status, new and re-treatment cases and resistance patterns to both first and second line anti-TB drugs. Besides, the technique was able to detect the NTM, a documented common co-infection in TB/HIV patients [31]. The NTM were detected in cases initially reported as sputa smear positive for TB, and interestingly, only one case was NTM in the HIV positive cases, while three of the NTM cases were all negative for HIV/AIDS.

## Conclusion

Overall, resistance to INH than RIF was commonly detected for the first line drugs using the MTBDR®plus assay, but no MDR-TB nor XDR-TB resistance patterns were detected in the studied population. However, MTBDRplus could be an alternative assay for the detection of NTMs which ordinarily can be missed from sputum smear evaluation.

## Supporting Information

S1 Table Results of gender against FLD. This set of calculations tested the association between male and female having first- line drug resistance TB.
(PDF)

S2 Table Results of age distribution against FLD. The table shows the distribution of age range against resistance to first line anti-TB drugs isoniazid and rifampicin.
(PDF)

S3 Table Results of MTB DR Plus. This is the confirmation for the sputum smear positive TB cases with MTB DR Plus test kit.
(PDF)

S4 Table Results of HIV status against MTBDR plus. Testing for HIV and TB co-infection among the study cases.
(PDF)

S5 Table Statistical significant correlations between HIV status and TB co-infection. Pearson Chi-Square analysis showed no significant statistical correlation between HIV status and TB co-infection since the corresponding p-value is greater than 0.05.
(PDF)

S6 Table Results of HIV status against FLD. This table indicates that majority of the study cases had unknown HIV status.
(PDF)

S7 Table Statistical significant correlations between HIV status and FLD. Pearson Chi-Square analysis, showing no significant statistical relationship between HIV status and FLD.
(PDF)

S8 Table Patients’ type with FL-DR TB. The table shows categorization of patients and resistance to first line drugs.
(PDF)

S9 Table Results of age distribution against SLD. Comparison between the distributions of age range against resistance to SLD.
(PDF)

S10 Table HIV status against SLD. A comparison between HIV status and SLD.
(PDF)

S11 Table Cumulative laboratory results of DR TB for both FLD and SLD. (PDF)

## Abbreviations

DR: Drug resistant
ETH: Ethambutol
FQ: Fluoroquinolones
RIF: Rifampicin
INH: Isoniazid
MDR: Multi drug resistant
NTM: Non tuberculosis mycobacterium
PTB: Pulmonary tuberculosis
TB: Tuberculosis
XDR: Extreme drug resistant
