# Supplementary material for: Prevalence of Drug Resistance Mycobacterium Tuberculosis among Patients Seen in Coast Provincial General Hospital, Mombasa, Kenya
Source: PLoS One. 2016 Oct 6;11(10):e0163994. doi: 10.1371/journal.pone.0163994 (PMC5053611; doi:10.1371/journal.pone.0163994)
Supplement: S1 Table — This set of calculations tested the association between male and female having first- line drug resistance TB. (PDF) [file pone.0163994.s001.pdf]

S1 table. Results of gender against FLD.

This set of calculations tested the association between male and female having first-line drug resistance TB

Study population		First line				Total
		MTB Negative	Fully susceptible	INH resistant	RIF resistant	
Sex	Male	4	164	5	1	174
	Female	3	78	3	0	84
Total		7	242	8	1	258
