# Supplementary material for: Prevalence of Drug Resistance Mycobacterium Tuberculosis among Patients Seen in Coast Provincial General Hospital, Mombasa, Kenya
Source: PLoS One. 2016 Oct 6;11(10):e0163994. doi: 10.1371/journal.pone.0163994 (PMC5053611; doi:10.1371/journal.pone.0163994)
Supplement: S2 Table — The table shows the distribution of age range against resistance to first line anti-TB drugs isoniazid and rifampicin. (PDF) [file pone.0163994.s002.pdf]

S2 table. Results of age distribution against FLD

The table shows the distribution of age range against resistance to first line anti-TB drugs isoniazid and rifampicin.

	1-10yr	11-20	21-30	31-40	41-50	51-60	61-70	71-80	Total
TB negative	0	1	1	5	0	0	0	0	7
FS	7	30	102	66	25	10	1	1	242
INH res	0	0	2	3	2	1	0	0	8
RIF res	0	0	1	0	0	0	0	0	1
Total	7	31	106	74	27	11	1	1	258
