# Supplementary material for: Prevalence of Drug Resistance Mycobacterium Tuberculosis among Patients Seen in Coast Provincial General Hospital, Mombasa, Kenya
Source: PLoS One. 2016 Oct 6;11(10):e0163994. doi: 10.1371/journal.pone.0163994 (PMC5053611; doi:10.1371/journal.pone.0163994)
Supplement: S3 Table — This is the confirmation for the sputum smear positive TB cases with MTB DR Plus test kit. (PDF) [file pone.0163994.s003.pdf]

S3 table. Results of MTB DR Plus results.

This is the confirmation for the sputum smear positive TB cases with MTB DR Plus test kit.

**Patient status**	**n (%)**
Negative	4 (1.6)
Positive	251 (97.3)
Neg. NTM	3 (1.1)
Total	258 (100)
