# Supplementary material for: Prevalence of Drug Resistance Mycobacterium Tuberculosis among Patients Seen in Coast Provincial General Hospital, Mombasa, Kenya
Source: PLoS One. 2016 Oct 6;11(10):e0163994. doi: 10.1371/journal.pone.0163994 (PMC5053611; doi:10.1371/journal.pone.0163994)
Supplement: S4 Table — Testing for HIV and TB co-infection among the study cases. (PDF) [file pone.0163994.s004.pdf]

S4 table. Results of HIV status against MTBDR plus results

Testing for HIV and TB co-infection among the study cases.

Study case		HIV status			Total
		Negative	Positive	Unknown	
MTBDR Plus	Negative	2(0.8%)	1(0.4%)	1(0.4%)	4
	Positive	94(36.4%)	46(17.8%)	111(43.0%)	251
	NTM	0(0)	1(0.4%)	2(0.8%)	3
Total		96	48	114	258
