# Supplementary material for: Prevalence of Drug Resistance Mycobacterium Tuberculosis among Patients Seen in Coast Provincial General Hospital, Mombasa, Kenya
Source: PLoS One. 2016 Oct 6;11(10):e0163994. doi: 10.1371/journal.pone.0163994 (PMC5053611; doi:10.1371/journal.pone.0163994)
Supplement: S5 Table — Pearson Chi-Square analysis showed no significant statistical correlation between HIV status and TB co-infection since the corresponding p-value is greater than 0.05. (PDF) [file pone.0163994.s005.pdf]

S5 Table. Statistical significant correlations between HIV status and TB co-infection.

Pearson Chi-Square analysis showed no significant statistical correlation between HIV status and TB co-infection since the corresponding p-value is greater than 0.05

	Value	df	Asymp. Sg. (2-sided)
Pearson Chi-Square	5.031	4	
N of Valid Cases	258		.968
