# Supplementary material for: Prevalence of Drug Resistance Mycobacterium Tuberculosis among Patients Seen in Coast Provincial General Hospital, Mombasa, Kenya
Source: PLoS One. 2016 Oct 6;11(10):e0163994. doi: 10.1371/journal.pone.0163994 (PMC5053611; doi:10.1371/journal.pone.0163994)
Supplement: S6 Table — This table indicates that majority of the study cases had unknown HIV status. (PDF) [file pone.0163994.s006.pdf]

S6 table. Results of HIV status against FLD.

This table indicates that majority of the study cases had unknown HIV status.

		First line result (DR)				Total
		Negative	Fully susceptible	INH res	RIF res	
HIV status	Positive	2(0.8%)	89 (34.1%)	5 (1.9%)	1 (0.4%)	96
	Negative	2 (0.8%)	44 (17.1%)	2 (0.8%)	0 (0)	48
	Unknown	3 (1.2%)	109 (42.6%)	1 (0.4)	0 (0)	114
Total		7	242	8	1	258
