# Supplementary material for: Prevalence of Drug Resistance Mycobacterium Tuberculosis among Patients Seen in Coast Provincial General Hospital, Mombasa, Kenya
Source: PLoS One. 2016 Oct 6;11(10):e0163994. doi: 10.1371/journal.pone.0163994 (PMC5053611; doi:10.1371/journal.pone.0163994)
Supplement: S7 Table — Pearson Chi-Square analysis, showing no significant statistical relationship between HIV status and FLD. (PDF) [file pone.0163994.s007.pdf]

S7 table. Statistical significant correlations between HIV status and FLD.

Pearson Chi-Square analysis, showing no significant statistical relationship between HIV status and FLD.

	Value	df	Asymp. Sig. (2-sided)
Pearson Chi-Square	5.623[a]	6	.467
Likelihood ratio	6.352	6	3.85
Linear-by-Linear Association	3.348	1	.067
N of valid Cases			

a. 9 cells 975.0%) have expected count less than 5. The minimum expected count is .19
