# Supplementary material for: Prevalence of Drug Resistance Mycobacterium Tuberculosis among Patients Seen in Coast Provincial General Hospital, Mombasa, Kenya
Source: PLoS One. 2016 Oct 6;11(10):e0163994. doi: 10.1371/journal.pone.0163994 (PMC5053611; doi:10.1371/journal.pone.0163994)
Supplement: S8 Table — The table shows categorization of patients and resistance to first line drugs. (PDF) [file pone.0163994.s008.pdf]

S8 table. Patients' type with FL-DR TB.

Legend: The table show categorization of patients and resistance to first line drugs.

		First line drugs				Total
		Negative	Fully sens	INH res	RIF res	
Study type	New cases	7 (2.7%)	237 (91.7%)	8 (3.1%)	1 (0.4%)	253
	Follow ups	0 (0)	1 (0.4%)	0 (0)	0 (0)	1
	Relapse	0 (0)	3 (1.2%)	0 (0)	0 (0)	3
	Defaulter	0 (0)	1 (0.4%)	0 (0)	0 (0)	1
Total		7	242	8	1	258
