# Supplementary material for: Prevalence of Drug Resistance Mycobacterium Tuberculosis among Patients Seen in Coast Provincial General Hospital, Mombasa, Kenya
Source: PLoS One. 2016 Oct 6;11(10):e0163994. doi: 10.1371/journal.pone.0163994 (PMC5053611; doi:10.1371/journal.pone.0163994)
Supplement: S9 Table — Comparison between the distributions of age range against resistance to SLD. (PDF) [file pone.0163994.s009.pdf]

S9 table. Results of age distribution against SLD

Comparison between the distributions of age range against resistance to SLD

Study population	1-10yr	11-20	21-30	31-40	41-50	51-60	61-70	71-80	Total
FS		13	47	14					74
ETH res				1					1
FQ res				1					1
Resistant to FQ, CAP, VIO, AMK, KAN			1						1
Resistance to CAP, VIO, AMK, KAN & ETH			1						1
Incomplete findings				2	2		1		5
**Total**	**0**	**13**	**49**	**18**	**2**	**0**	**1**	**0**	**83**
