# Supplementary material for: Prevalence of Drug Resistance Mycobacterium Tuberculosis among Patients Seen in Coast Provincial General Hospital, Mombasa, Kenya
Source: PLoS One. 2016 Oct 6;11(10):e0163994. doi: 10.1371/journal.pone.0163994 (PMC5053611; doi:10.1371/journal.pone.0163994)
Supplement: S10 Table — A comparison between HIV status and SLD. (PDF) [file pone.0163994.s010.pdf]

S10 table. HIV status against SLD.

A comparison between HIV status and SLD

		Incomplete findings	Second line drugs					Total
			Fully susceptible	FQ res	ETH res	Res to CAP, VIO, AMK, KAN, ETH	Res to FQ, CAP, VIO, AMK, KAN	
HIV status	Negative	2 (2.4%)	27 (32.5%)	1 (1.2%)		1 (1.2%)		31 (37.3%)
	Positive		15 (18.1%)					15 (18.1%)
	Unknown	3 (3.6%)	32 (38.6%)		1 (1.2%)		1 (1.2%)	37 (44.6%)
Total		5	74	1	1	1	1	83
