# Supplementary material for: Prevalence of Drug Resistance Mycobacterium Tuberculosis among Patients Seen in Coast Provincial General Hospital, Mombasa, Kenya
Source: PLoS One. 2016 Oct 6;11(10):e0163994. doi: 10.1371/journal.pone.0163994 (PMC5053611; doi:10.1371/journal.pone.0163994)
Supplement: S11 Table — (PDF) [file pone.0163994.s011.pdf]

S11 table. Cumulative laboratory results of DR TB for both FLD and SLD

FLD	SLD	
	Mono resistance	Poly-resistance
Susceptible to both ETH and RIF= 242 (93.8%)<br>RIF susceptible= 8 (3.1%)<br>INH susceptible =1 (0.4%)<br>Negative result = 7 (2.7%)	To ETH= 1 (1.2%)<br>To FQ=1 (1.2%)	To FQ, CAP, VIO, AMK, KAN=1 (1.2%)<br>(But was fully susceptible in the 1st line)
	Fully susceptible= 74 (89.2%)	
	Incomplete= 5 (6.0%)	
Hence no MRD	Hence no XDR	
**Total= 258**	**Total= 83**	
